# Evaluating mobile apps for sun protection: content analysis and user preferences in a two-part study

**DOI:** 10.1080/21642850.2025.2456659

**Published:** 2025-01-27

**Authors:** Angela M. Rodrigues, Faye L. Doughty, Caroline Charlton, Sarah Woodhouse, Elizabeth Sillence

**Affiliations:** Department of Psychology, Northumbria University, Newcastle upon Tyne, UK

**Keywords:** Behaviour change technique, mobile phone application, sun protection, digital health, skin cancer prevention

## Abstract

**Background:**

Sunburn and intermittent sun exposure elevate melanoma skin cancer risk. Sun protection behaviours, including limiting sun exposure, seeking shade, wearing protective gear, and using sunscreen, help mitigate excessive sun exposure. Smartphone apps present a promising platform to enhance these behaviours.

**Objective:**

Part 1 aimed to analyse and evaluate the content of mobile apps that encourage sun protection behaviours, focusing on features, and behaviour change techniques (BCTs). Part 2 explored user preferences and usability post-initial use and two weeks later.

**Results:**

Part 1 identified 1294 apps; after applying exclusion criteria, 87 apps were downloaded, with 48 included for analysis. The apps presented opportunities for enhancement in their theoretical and evidence basis, and visualisations use (e.g. UV-index). The apps mapped across a total of 12 BCTs (*M* = 1.71, *SD* = 1.07; range = 0–5). The most frequently identified BCTs were ‘instruction on how to perform behaviour’ (65%), ‘information about health consequences’ (29%), and ‘prompts/cues’ (27%). In Part 2, participants favoured features supporting knowledge and ease of use. Participants expressed a preference for apps that are free of paid features, advertisements, and external purchases. Tailored advice (e.g. location, skin type) was deemed crucial, particularly for initial exposure. Proactive features integrating behavioural, personal, and contextual information for adaptive and just-in-time sun protection advice were seen as essential for sustaining engagement.

**Conclusions:**

Sun protection apps emphasizing knowledge, ease of use, tailored advice, and proactive features are likely to encourage sustained engagement. Suggestions for optimising current and future sun protection apps are provided.

## Introduction

Skin cancer is common, with a lifetime risk of 1 in 5 for the UK population (Venables et al., 2019) and it is increasing in incidence. It is estimated that the majority of skin cancers are due to excess ultraviolet exposure (Brown et al., 2018), from the sun and from sunbeds (Boniol et al., 2012). Risk factors include fair skin that burns easily, a family history of melanoma, the presence of multiple naevi, and significant sun damage (Usher-Smith et al., 2017). These factors highlight the vulnerability of specific groups within the population, particularly those who experience frequent or intense UV exposure.

It is estimated that 86% of skin cancer cases could be prevented, saving £100 million annually in the UK (Robertson & Fitzgerald, 2017). Skin cancer prevention initiatives have been shown to have both cost and health benefits at a population level (Collins et al., 2024). A recent systematic review reports highly favourable returns on investment for prevention, ranging from US$0.35 to $1 spent and up to €3.60 per €1 invested (Collins et al., 2024). Given the significant potential for prevention, adopting consistent sun protection practices to reduce excess sun exposure becomes essential. These behaviours include reduce midday sun exposure, seek shade, wear protective clothing, hats, sunglasses, use sunscreen, and avoid artificial tanning devices like sunbeds (WHO, 2024).

Digital health solutions, such as mobile phone applications, offer a promising avenue for reaching large segments of the population and promoting healthy behaviours (Abroms et al., 2012). Studies have shown that apps can increase sun protection behaviours, presenting a valuable opportunity for public health (Buller et al., 2015). User engagement, a key factor in the sustained use of health apps, is defined as positive experiences that encourage ongoing usage over time (Kim & Baek, 2018). Engagement with digital health interventions has been linked to successful behaviour change (Yardley et al., 2016), highlighting the importance of creating apps that appeal to users. Studies have shown that many health apps are discontinued after limited use, often due to poor user-friendliness (Baumel et al., 2019; Zhao et al., 2016). Addressing app functionality and incorporating desirable features – such as user-friendly interfaces, detailed information, and personalised experiences – can enhance user satisfaction and reduce abandonment rates (Murnane et al., 2015; Zhao et al., 2016). To maximize behaviour change, health apps should focus on increasing motivation and self-efficacy while incorporating social networking features (Fitzgerald & McClelland, 2016). Therefore, to enhance engagement with sun protection apps, it is crucial to understand not only the specific behaviour change techniques and features being utilised but also user perspectives on their usability. By doing so, we can design more effective, engaging tools that promote long-term health benefits.

A previous systematic review highlighted the opportunity for improved reporting of sun protection interventions, which remains a key area for progress (Rodrigues et al., 2013). By enhancing the clarity and consistency in describing how behavioural interventions are derived, greater transparency and reproducibility can be achieved, leading to more effective and scalable interventions. When seeking to understand how behaviour can be influenced, the use of theory-based classification systems (taxonomies) has proven valuable in identifying and categorising behaviour change techniques (BCTs) (Michie et al., 2013). Different theories, such as Theory of Planned Behaviour (Ajzen, 1991), Social Cognitive Theory (Bandura, 1986), and the Health Action Process Approach (HAPA; (Schwarzer, 2008)), as well as appearance-based interventions (Mahler, 2018), have been proposed to explain behaviour change applied to sun protection, photoprotection, and skin cancer prevention (Craciun et al., 2011; Hacker et al., 2018; Janda et al., 2013; White et al., 2015). These theories emphasise constructs such as motivation, perceived risk, self-efficacy, outcome expectancies, and planning. Including these theoretical perspectives provides a foundation for understanding and categorising BCTs. BCTs, defined as ‘active components of an intervention designed to change behaviour’, are designed to impact a variety of psychological processes and mechanisms essential for behaviour change (Michie et al., 2013). For example, techniques like action planning and goal setting align closely with constructs emphasised in the HAPA model (Schwarzer, 2008), while strategies focusing on self-monitoring or feedback on behaviour can be linked to mechanisms within Social Cognitive Theory (Bandura, 1986). By mapping these theoretical constructs to specific BCTs, researchers can systematically design and evaluate interventions. This categorisation provides a practical framework to translate theoretical constructs into actionable intervention features.

Previous studies have demonstrated the effectiveness of specific BCTs in promoting sun-protection behaviours by providing clear information about health and appearance-related consequences of sun exposure, including the risks of photoaging (e.g. ultraviolet photographs), challenging the appeal of tanning, and fostering social support (Rodrigues et al., 2013; Sheeran et al., 2020). Social norm-based approaches, such as facilitating social comparisons, as well as practical strategies like providing free sunscreen, modelling correct application, and using reminders, have also been shown to enhance sun-protection practices (Sheeran et al., 2020). Additionally, visualisation techniques, such as detailed UV index information, may further support health-related behaviour change by reinforcing sun protection messages (Heckman et al., 2019; Hollands et al., 2022). These findings underscore the potential of well-designed interventions to significantly enhance sun protection behaviours.

The aim of this study was twofold: Part 1 focused on analysing and evaluating the content of mobile phone apps designed to encourage sun protection behaviours (e.g. limit midday sun, seek shade, wear protective clothing, hats, sunglasses, apply sunscreen) available on Google Play and the iOS App Store, while Part 2 explored user feedback and interaction with these apps. Specifically, we aimed to answer the following research questions: (a) What types of features and behaviour change techniques are incorporated into sun protection apps? (b) How do potential users engage with sun protection apps, and what factors influence their reactions and continued use? By first understanding the features and content elements in Part 1 and then exploring user experiences in Part 2, we provide a comprehensive understanding of the apps’ usability. This progression from content analysis to user feedback allows us to understand not only what features are included but also how they are perceived and used in practice. This combination is necessary to evaluate both the technical and experiential aspects of sun protection apps, and to provide details on how app design influences user engagement and behaviour change.

## Methods

### Part 1

#### Design

In Part 1, we conducted a content analysis of mobile apps designed to promote sun protection behaviours. The analysis involved evaluating app features, and coding behaviour change techniques (BCTs) according to the BCT Taxonomy v1 (Michie et al., 2013). We conducted a systematic search to identify apps and used a systematic coding framework to assess the inclusion of various BCTs and features.

#### Search strategy and data extraction

The sun protection apps were identified by searching the UK versions of Google Play and the IOS’s app stores throughout March and April 2020. Both stores were searched using 9 search terms: ‘sun protection’, ‘UV index’, ‘sunscreen’, ‘sunburn’, ‘skin ageing’, ‘tan’, ‘suntan’, ‘sunlight’, and ‘sunbathing’. For the Google Play store, we selected the top 100 apps from the initial results for each search term, totalling 900 results. For the iOS App Store, we included apps based on the search results, with most terms yielding fewer than 100 apps, totalling 394 results. This approach mirrors methods from previous studies that focused on popular apps by examining top-ranked results in app stores (Azar et al., 2013; Breton et al., 2011; Pagoto et al., 2013). Apps were only included if they were ‘free of charge’. We focused exclusively on free at the point of download apps to ensure that the analysis covered widely accessible sun protection solutions, aligning with our goal to evaluate apps available to a broader user base.

**Figure 1.**
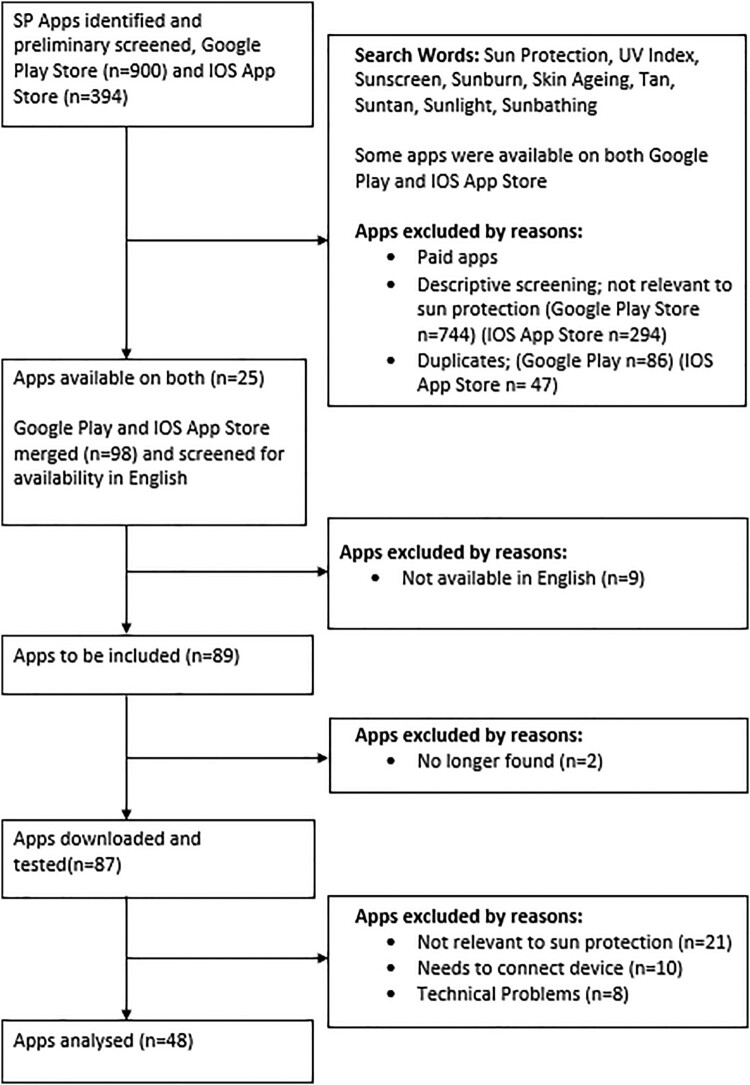
Flow diagram of apps selected for coding.

Exclusion criteria were applied to the identified apps: apps were removed if they required payment to download, were not relevant to sun protection, or were duplicates. Of the 98 apps remaining, 9 were not available in English and 2 were no longer found, this left 87 apps which were downloaded and tested. Further apps were excluded once they had been downloaded, 21 were found to not be relevant to sun protection, 10 required a connecting device, and 8 showed technical problems (see Figure 1). The sun protection apps were coded on an iPhone using IOS 13.5.1 and on a Samsung using Android 7.0. The final remaining 48 apps were analysed and coded for features, and inclusion of BCTs.

#### Features assessment

The feature assessment was developed based on assessments used in previous studies (Chen et al., 2015; Zhao et al., 2016), and supplemented with insights from sun protection literature (Rodrigues et al., 2013; Sheeran et al., 2020). Each of the 48 apps was evaluated based on a set of key features that are relevant to sun protection interventions. These features included: theory-based content, evidence-based practices, behaviour tracking, visualisation tools, social media integration, location-based features, notifications, and personalisation/tailoring options. As done in previous studies, the total score reflects the presence of these features, plus the use of specific evidence-based BCTs (Chen et al., 2015; Lyons et al., 2014), with a maximum possible score of 30. For the relevant scoring system and additional detail on features assessment, please see Table 1. For more information regarding the development of the criteria and scoring system, please see supplemental material Table 1.

**Table 1.** Features Assessment criteria and scoring system.

Features	Score (total = 30)
Theory	1
Evidence	1
Tracking type - Sunscreen - Sunbathing - UV Index	1 (per tracking type)
Visualisation Features - Photo Uploads - UV Photos - Skin Aging (face) - UV Index Visualisations - Mole Visualisations	1 (per feature)
Social Media (Yes/No)	1
Location Features (yes/no) - Environment type - Sun/Clouds - UV Forecast	1 (Overall: if any features were present)
Notifications (yes/no)	1
Tailoring (yes/no) - Location (adjustable, current) - Skin colour - Tan - Burn - Sensitivity - UV Exposure - Clothing - Freckles - Hair colour - Eye colour - SPF - Height - Weight - Age - Gender	1 (Overall: if any features were present)
Presence of evidence-based BCTs (*n* = 16)	1 (per BCT)

#### Behaviour change technique coding

An international taxonomic classification system for BCTs (BCT Taxonomy v1) (Michie et al., 2013) containing 93 BCTs was used to code the BCTs included in the sun protection apps. The manual included definitions and examples of each BCT. One researcher (CC) used the coding manual to independently code all 48 apps using the BCT Taxonomy v1. To ensure reliability, one researcher (AMR) randomly selected 10% of the apps (5 apps) for double coding by another trained researcher. Both researchers had prior training in BCT coding and participated in an initial calibration session to ensure consistency in applying the coding framework. The BCT coding agreement was assessed by comparing the two sets of coded data. Any discrepancies identified were discussed thoroughly between the two researchers, and consensus was reached through discussion. Final agreement was defined as both coders fully agreeing on all BCT codes for the sample apps. Once full agreement was reached, the researcher proceeded with coding the remaining apps independently.

Of the 93 BCTs described in the BCT Taxonomy v1 (Michie et al., 2013), 16 have been found to be frequently used in health-related apps (Zhao et al., 2016) and interventions promoting sun-protection (Rodrigues et al., 2013; Sheeran et al., 2020). To establish the extent to which these BCTs were included in the reviewed apps, 1 coder (CC) and an independent behaviour change expert (AMR) mapped the 16 frequently used BCTs to the BCT Taxonomy v1. This allowed us to determine the frequency of those BCTs in the reviewed apps.

In summary, while the feature assessment highlights the key functional components of the apps (e.g. notifications, personalisation), the BCT coding identifies how those functionalities translate into active ingredients designed to drive behaviour change.

### Part 2

#### Design

Part 1 established a baseline understanding of app content, which informed the selection of apps and focus areas for part 2. In part 2, we employed a two-week longitudinal design to assess user interactions with sun protection apps through think-aloud methodology and semi-structured interviews. This duration was chosen based on the observation that apps are often abandoned after about ten uses, with typical usage occurring several times per week (Jaspers et al., 2004; Robbins et al., 2017). Participants performed tasks using the think-aloud method to provide real-time feedback on app features (Jaspers et al., 2004; Yardley et al., 2016). This was complemented by semi-structured interviews to gather detailed user experiences. Both methods aimed to explore usability and user preferences. Ethical approval for this study was granted from Faculty of Health Sciences ethics committee at Northumbria University (Ref: 49008).

#### Participants

Participants were recruited in the UK from a nonclinical population who had access to a mobile device between July and November 2022. Participants had to be at least age 18 to take part and could attend an initial interview at Northumbria University (Newcastle Upon Tyne, UK), followed by an online interview two weeks later. None of the participants had used a sun protection app before. Participants were asked if they booked or intend to book a holiday in a hot location (UK or abroad) during the initial questionnaire, of which 15 had. 23 people registered interest in the study via the initial questionnaire, yet three people did not answer. Thus, a total of 20 participants were recruited and attended initial interviews. 17 of the 20 participants attended the follow-up interview. All participants provided online informed consent to take part.

#### Sampling

Participants were recruited through posting an advertisement to the pre-test survey via social media platforms such as Facebook, LinkedIn, and Instagram. The study aims and exclusion criteria were outlined in the social media posts and participant information sheet. To be eligible, participants had to be aged 18 or older and available to attend on the specified study appointment date. Participants received a travel-size sunscreen bottle as a token of appreciation for their participation.

#### Procedure

Participants completed a pre-test survey and were then randomly assigned to 1 of 3 top-ranked sun protection apps via Qualtrics randomisation. Due to technical issues, seven participants allocated to app A were reassigned to app B or C. In total, four participants used app A, eight used app B and eight used app C. Prior to the initial interview, all participants provided their informed consent. The initial interview required participants to explore the app they had been allocated whilst thinking aloud. They were then asked semi-structured interview questions (Supplemental materials). The questions asked if the app was easy to download and set up, and if the app was engaging. After taking part in the initial interview, participants were asked to continue using the app for 2 weeks and were given no specific instructions on app usage. After 1 week, participants were contacted to ensure usage was still taking place. They were then contacted again before week 2 to arrange an online follow-up interview (Supplemental materials) and complete the post-test survey. The follow-up interview covered overall opinion of the app, specific app functionalities, app engagement, and further improvements. While we did not systematically track weather or UVI conditions during the two-week study period, retrospective weather for the study period was explored to provide further context, with temperatures in Newcastle (UK) as follows: 18°C in July; 17.5°C in August; 15.5°C in September; and 12.5°C in October.

#### Measures

The pre-test survey included demographics questions (e.g. gender identity; age), smartphone use questions (e.g. phone type), skin type assessment (I – Burns easily, never tans to VI – Never burns, tans profusely) and 7 self-reported items on sun-protection, assessing exposure times, sunscreen, hat, t-shirt and sunglasses usage, and seeking shade (Glanz et al., 2008). Participants were also asked if they had booked or intended to book a holiday in a hot location (UK or abroad), but follow-up questions regarding the timing or location of these holidays were not included in the post-survey.

The post-test survey included the same items on sun protection behaviours and also asked about app features based on the MARS scale, which is designed to assess the quality of mobile apps (Terhorst et al., 2020). A previous evaluation of the MARS demonstrated good concurrent validity (correlation with ENLIGHT; ps < .05), good to excellent reliability (Omega 0.79–0.93) and high objectivity (ICC = 0.82) (Terhorst et al., 2020). The MARS was developed to be an easy-to-use, objective, and validated tool to evaluate the quality of mobile apps across multiple dimensions. The scale also includes a subjective assessment of mobile apps that we decided to include given that participants were actual users of the app. There was a total of 21 items on the questionnaire, assessed through a 5-point Likert scale from strongly disagree to strongly agree. Both surveys were built using Qualtrics software.

#### Mobile apps

Part 1 provided foundational data on app content, while Part 2 built on this by exploring user interactions and feedback. Based on Part 1, the top-ranked apps for sun protection were selected for Part 2. However, due to the unavailability of the third and fourth highest-rated apps (SunSafe and UV Index Forecast) on the app store at the time of Part 2, IONIQ Skincare, the next highest-ranked app, was included. The apps randomly allocated to participants were: (A) *IONIQ Skincare*, (B) *REAPPLY;* and (C) *QSun.* The researchers had no association with the apps.

#### Treatment of data

SPSS Version 26 was used to calculate descriptive statistics for participants survey responses. Audio recordings, from the interviews, were transcribed using verbatim transcription to create qualitative data. Participants were given identifiers to protect their personal data and ensure anonymity.

The data were analysed using framework analysis to identify barriers and facilitators of sun protection apps use and followed the stages of familiarization, identification of thematic framework, indexing, charting, mapping, and interpretation (Gale et al., 2013). Indexing was completed by (FD) using QSR NVivo 12. The coding framework was refined iteratively through repeated discussions with the first author (AMR). The data were charted, and the responses were grouped according to the finalised thematic framework. During mapping and interpretation, the grouped data were examined by (FD) to identify patterns. Initial and follow-up interviews were analysed separately.

### Ethics statement

Full institutional ethical approval was received for this research.

- Institutional Review Board Statement: The study was conducted in accordance with the Declaration of Helsinki and was approved by an Institutional Review Board/Ethics committee. See details under Methods. ✓
- The study received an exemption from an Institutional Review Board/Ethics committee; See details under Methods.

## Findings

### Part 1

#### Features assessment

The features assessments for each app are summarised within Table 2. Overall, SunSafe and REAPPLY: Sunscreen Timekeeper had the most features, with a score of 10 out of 30. The overall mean features score for the apps was 5 features. Out of the 48 apps assessed, 0 apps included theory, only 1 app included evidence, 10 apps included at least 1 visualisation feature, 29 apps included at least 1 feature that could be tailored towards the user, 1 app incorporated social media features, 38 apps included at least 1 location feature and finally, 41 apps had the ability to send notifications. Apps which included a higher number of effective BCTs were found to score higher overall within the features assessment (see Table 4 for details on BCTs identified across the apps).

**Table 2.** Features assessment for apps.

App	Total Score
SunSafe	10
REAPPLY: Sunscreen Timekeeper	10
Qsun – Vitamin D & UV Tracker	9
SunBlock – Protect your skin	8
IONIQ Skincare	7
Sunscreen – Protect your skin	7
Sunscreen Helper	7
SunSense	7
UV Index Forecast	7
UV Safe – Sun Protection	7
UVLens – UV Index	7
Uvlower	7
AM Sun Expert	6
DermlA	6
Healthy Sun – safe tan	6
My SKIN TRACK UV	6
Save your skin	6
Suncap – UV Index	6
Sunface – UV – Selfie	6
UV Index Global	6
GlobalUV	5
InfoSun	5
Solarize	5
SunDay: Vitamin D & UV Tracker	5
Sunscreenr Mobile	5
UV Index Now – UVI Mate	5
UV Index Widget	5
UV Notifier	5
UV Skin Protection	5
UV-INDEKS	5
IndiceUV	4
Mollie’s fund	4
My UV Index	4
Sun Visor	4
Sunny@SG	4
UV Index	4
UV Index by dnzh	4
UV-Index	4
Uvisio	4
OzSun UV Alert	3
Sunbeam: UV Forecast	3
UV Index – App	3
VBS UV Index Monitor	3
How to prevent a sunburn	2
SkinSmart	2
Sunbathing & UV	2
Wear Sunscreen	2
Cache Cache Soleil	1

^a^ Bold line indicates median features assessment cut off.

#### Behaviour change techniques in sun protection apps

Out of a potential 93 BCTs within BCT Taxonomy v1, the mean number of BCTs found in sun protection apps was 1.71 (SD 1.07, MD 2). There were 6 apps in total that did not display any BCTs. The maximum number of BCTs present was 5 (*n* = 1), and a total of 12 BCTs were displayed at least once within all identified apps.

The frequency of the BCTs present in the sun protection apps is shown in Table 3. The most frequent BCTs were ‘instruction on how to perform the behaviour’ (64.58%, 31/48), ‘information about health consequences’ (29.16%, 14/48), ‘prompts/cues’ (27.08%, 13/48), ‘feedback on outcomes(s) of behaviour’ (14.58%, 7/48), and ‘feedback on behaviour’ (12.5%, 6/48).

**Table 3.** Frequency of BCTs displayed in sun protection Apps (*N* = 48 apps).

BCT	*N* (%)
4.1 Instruction on how to perform the behaviour	31 (64.58)
5.1 Information about health consequences	14 (29.16)
7.1 Prompts/cues	13 (27.08)
2.7 Feedback on outcome(s) of behaviour	7 (14.58)
2.2 Feedback on behaviour	6 (12.50)
2.3 Self-monitoring of behaviour	4 (8.33)
9.1 Credible source	2 (4.16)
1.1 Goal setting (behaviour)	1 (2.08)
1.4 Action planning	1 (2.08)
2.1 Monitoring of behaviour by others without feedback	1 (2.08)
2.4 Self-monitoring of outcome(s) of behaviour	1 (2.08)
5.3 Information about social and environmental consequences	1 (2.08)

Out of the total 16 BCTs found to be effective in other sun protection interventions, 7 of these were also present in the sun protection apps. Of these 16, the 7 included were ‘instruction on how to perform the behaviour’ (64.58%, 31/48), ‘information about health consequences’ (29.16%, 14/48), ‘prompts/cues’ (27.08%, 13/48), ‘feedback on behaviour’ (12.5%, 6/48), ‘self-monitoring of behaviour’ (8.33%, 4/48), ‘credible source’ (4.16%, 2/48), and ‘information about social and environmental consequences’ (2.08%, 1/48). Nine of the 16 BCTs found to be effective in other health behaviour change interventions were not present in any of the sun protection apps, ‘social comparison’, ‘information about others’ approval’, ‘adding objects to the environment’, ‘demonstration of behaviour’,‘salience of consequences, ‘future punishment’, ‘framing/reframing’, ‘social support (unspecified)’ and ‘pros and cons’. See Table 4 for BCT mapping of apps (table only includes apps which mapped the number of BCTs above the median [*n* = 2]). See supplemental materials for full BCT mapping of all apps included within the analysis.

**Table 4.** Frequency of Evidence-Based BCTs displayed in sun protection apps.

Behaviour change techniques^a^ Sun protection App^b^	Number of BCTs^c^	4.1^d^	5.1^d^	7.1^d^	2.2^d^	2.3^d^	5.3^d^	9.1^d^	2.7	1.1	1.4	2.1	2.4
REAPPLY: Sunscreen Timekeeper	5	X	X		X		X	X					
Qsun – Vitamin D & UV Tracker	4	X	X	X					X				
UV Index Forecast	4	X	X	X					X				
IONIQ Skincare	3	X	X	X									
UVLens – UV Index	3			X	X	X							
SunSafe	3	X	X	X									
SunBlock – Protect your skin	3			X	X			X					
Sunscreen Helper	3	X	X	X									
Solarize	3	X	X	X									
Uvlower	3			X	X	X							
Sunface – UV – Selfie	2	X	X										
My SKIN TRACK UV	2	X	X										
UV Skin Protection	2	X	X										
Save your skin	2	X	X										
SkinSmart	2									X			X
Suncap – UV Index	2	X	X										
SunDay: Vitamin D & UV Tracker	2	X										X	
Sunscreen – Protect your skin	2	X	X										
SunSense	2	X									X		
UV Index Global	2	X	X										
UV Index Now – UVI Mate	2			X					X				
UV Index Widget	2	X							X				
UV Notifier	2	X							X				
UV Safe – Sun Protection	2	X		X									
UV-INDEKS	2	X							X				
Wear Sunscreen	2	X			X								
AM Sun Expert	2					X			X				
How to prevent a sunburn	2	X	X										
	**Total**	22	15	11	5	3	1	2	7	1	1	1	1

^a^ 4.1: Instruction on how to perform a behaviour, 5.1: Information about health consequences, 7.1: Prompts/cues, 2.7: Feedback on outcomes of behaviour, 2.2: Feedback on behaviour, 2.3 Self-monitoring of behaviour, 9.1 Credible source, 1.1 Goal setting (behaviour), 1.4 Action Planning, 5.3 Information about social and environmental consequences, 2.1 Monitoring of behaviour by others without feedback, 2.4 Self-monitoring of behaviour.

^b^ Sun protection apps ordered by frequency of behaviour change techniques (*n* = 48).

^c^ BCTs included in sun protection apps ordered by frequency (high to low) (*n* = 12).

^d^ BCTs shown to be the most effective in the literature.

Considering both the features and BCT assessments, REAPPLY: Sunscreen Timekeeper and Qsun – Vitamin D & UV Tracker emerged as the top-rated apps, demonstrating an inclusion of user-friendly features and evidence-based BCTs.

### Part 2

#### Descriptive statistics

A total of 20 participants took part in the part 2. The age of participants ranged from 18 to 53 years old (*M* = 26.36, *SD* = 10). Five participants identified as male (*M* age = 26.40, *SD* = 14.89); and 15 participants identified as female (*M* age = 26.35, *SD* = 8.71). The sample included a high proportion of fair-skinned participants (*n* = 17; 77%) who reported a tendency to burn (*n* = 15; 68%), aligning with key risk factors for skin cancer. For those planning an upcoming holiday (*n* = 15; 68%), the most reported holiday destination was Spain (*n* = 10; 67%). Among the 23 participants who completed the baseline questionnaire, 20 participants (20/23, 87%) completed the initial interview and 15 completed the follow-up survey (15/23, 65%).

At follow-up, sun exposure was high at weekends (81%), with a quarter of participants intentionally seeking a tan (27%). Sun protection was practiced by approximately half of the participants (range 40% [t-shirt use]-67% [sunscreen use]), except hat use which was low (20%).

#### MARS analysis

The follow-up survey asked participants to rate each app using the MARS scale. All three apps obtained a MARS score of over 3 points (acceptable quality) (Table 5). The MARS app quality mean score was 3.7 (REAPPLY), 3.6 (QSun), and 3.3 (IONIQ), which was slightly above the minimally acceptable quality for apps. The MARS subjective quality mean score was below the minimally acceptable quality with QSun scoring the highest (2.4). The MARS score for each app was higher than subjective quality score.

**Table 5.** Mean scores (users) on the Mobile App Rating Scale (MARS) rating categories for sun protection apps.

Sun Protection App	Mean MARS Score	Engagement Mean	Functionality Mean	Aesthetics Mean	Information Mean	Subjective Quality Mean
IONIQ	3.3	2.5	3.8	3.8	3.8	2
Qsun	3.6	3.2	3.9	3.5	3.8	2.4
REAPPLY	3.7	3.3	4	3.9	3.8	2.2

#### Factors influencing the uptake of sun protection apps

Eleven themes were found in relation to the usefulness of sun protection apps during initial interviews: (1) improving knowledge; (2) ease of use (e.g. quick set-up, lay language); (3) availability of location monitoring; (4) providing notifications (e.g. sunscreen reminders); (5) visual appeal (e.g. graphics and design); (6) availability of personalisation (e.g. skin type); (7) dissatisfaction with extra costs (e.g. premium subscriptions) and (8) technical issues (e.g. errors); (9) app usage associated with weather conditions; (10) need for inclusivity (i.e. gender) and (11). accessibility (i.e. font size). Table 6 provides an overview of themes related to participants’ first experience with using the app after using it for a two-week period (see Table 6). Similar themes were found in relation to usefulness of sun protection apps during follow-up interviews (after 2-week usage); however, some were expressed differently. During initial interviews, participants preferred apps which improved their knowledge of sun protection and used credible and accessible sources. At follow-up, participants expressed the same views, but this should be continued over time, to engage users, by using quizzes and short bursts of information. Knowledge shaping was also closely tied to UVI information provided by the app, which participants perceived as helpful to understand when sun protection is recommended. Participants continued to suggest that apps should have a simple account set up, adding that difficult apps can deter use over time (i.e. it should be simple to log back into apps). Upon using the app, participants reflected on the benefit of location monitoring and UV levels information, indicating that this feature could be useful to plan activities outside avoiding midday UV levels and potentially sun damage. As identified in Part 1, apps like REAPPLY and Qsun included user-friendly features such as sunscreen use tracking, UV forecast, tailoring and notifications. In Part 2, participants particularly valued these features, with some noting that they helped them better regulate sun protection routines.

**Table 6.** Themes found in relation to initial and follow-up interviews with participants.

Theme	Description	Initial interview quote	Follow-up quote
Shaping Knowledge	Participants highlighted that apps need to provide information from credible sources. (Facilitator)	‘It has the sources where it has come from. So, it’s not just random information and is backed up with evidence.’ – P6.	‘When I went on the (app) each day it told me the sun protection factor I should be using, which I found quite useful. It also told me how many minutes of sun exposure would lead to skin damage … It’s important to know because it might not be like a sunny day or hot, but you do still need the protection.’ – P4.
Ease of Use	Participants expressed the importance of a user-friendly interface, quick set-up, and use of lay language. (Facilitator)	‘I like the lay language because it is very easy to understand.’ – P3. ‘It was easy to download. Easy to set up, yes it was. It’s a typical set up for many apps really’ – P7.	‘I feel like it was very easy to set up. It didn’t ask you to create an account which just made like the set up and download process like quite smooth and it just went straight into the app.’ – P1.
Location Services	Participants expressed a preference for apps which provided UV and weather monitoring in their exact location. (Facilitator)	‘I think the feature that shows the weather outside and the high UV level is something useful.’ – P4.	‘I would say the advantages would be knowing which hours to avoid the sun and the weather in the area. It’s a quick way of going onto the app and seeing the hours of when it’s going to be sunny and when you may burn.’ – P11.
Notifications	Participants emphasised using notifications to remind users of when to apply their sunscreen and UV/weather changes. (Facilitator)	‘ I like that it sends notifications because if you forget, and you have your phone, it will always remind you when to put it on [sunscreen]’ – P6.	‘Having the notifications on. The one that gives you one every 40–90 minutes with the timer on. I’d say that was the most useful one.’ – P13.
Visual Appeal	Participants expressed that apps need to be appealing with a consistent app design and graphics. (Facilitator)	‘It looks appealing which was the biggest engagement factor for me’ – P3.	‘It’s a good app. It’s colourful too which is always a good thing.’ – P7.
Personalisation	Participants preferred information based on their skin-type and individual measurements. (Facilitator)	‘I think that this quiz has been very helpful to figure out my skin type’ – P3.	‘I thought that when it tailors it to your skin type. For example, mine was dry skin it would tell you that you should use products with vitamin E and aloe vera … I guess that would encourage me to be more aware of my skin protection habits.’ – P18.
Extra Costs	Participants did not prefer apps which contained extra purchases. (Barrier)	‘Not a lot of it is free so. I don’t think it’s good because you must pay to get all the good features’ – P8.	‘There was a lot of paid features which I found quite irritating. I felt like there was more paid features than non-paid features. There was more than half of the app which was paid. So, I think I’d change that.’ – P19.
Technical Issues	Participants encountered some technical problems, included broken links. (Barrier)	‘Oh, there’s an error on the app. When I clicked on what is UVAUVB it said error this domain isn’t connected to a website yet’ – P6.	‘When I created my account, it kept me logged in for a couple of days, then it logged me out. I tried to sign in using the same details, but it didn’t work. So, I had to create another account with a different email and password.’ – P16.
Location of Use	Participants expressed that engagement with the app is dependent on the location and weather conditions. (Barrier)	‘Looking at the app, I would say it’s useful. But from a perspective, being in England, I wouldn’t use it personally because I never wear, maybe in the summer I might wear sunscreen. If I was on holiday, I would use it though.’ – P16.	‘But it was raining and there was no sun so I thought it wasn’t something I’d personally pay for. We live in England so there’s basically no sun to the point where you’d get burned as the temperature is too cold.’ – P16.
Inclusivity	Participants highlighted a need for inclusivity, e.g. non-binary gender options. (Barrier)	‘There is no option for diverse, which I personally do not need, but I always like the option to be there. I know some people would like to have it.’ – P3.	–
Accessibility	Participants expressed that settings are needed to customise apps e.g. larger font size. (Barrier)	‘The font isn’t usually that tiny and most apps update to make it more accessible for reading.’ – P12.	–

Interestingly, some participants continued to highlight that apps would be more useful in warmer and sunnier climates (i.e. on holidays), whereas the apps were not useful in colder locations. Participants frequently cited the UK’s unpredictable weather as influencing their sunscreen use, with some expressing a lack of need for sun protection on cloudy or colder days.

## Discussion

### Principal findings

The findings from this study, which comprised a comprehensive analysis of sun protection apps in Part 1 and an exploration of user feedback and interactions in Part 2, provide valuable information into both the content and user experience of these digital health tools. The features assessment in Part 1 revealed opportunities for enhancement in the theoretical foundation and evidence-based content of the apps. Many of the apps have the potential to enhance user experience by incorporating visualisations, particularly those based on the UV index or UV photos. Additionally, the use of tailoring in just under half of the apps analysed presents an opportunity to further improve engagement. Overall, the apps mapped across a total of 12 behaviour change techniques (BCTs), ranging from 0 to 5 BCTs identified within the apps. There is no universally accepted threshold for what constitutes an effective health app; however, evidence from other health domains suggests that apps with a greater variety of features, personalised options, and high user engagement are generally more effective. For example, more popular menopause apps tend to have more features (Sillence et al., 2023), and smoking cessation apps show a positive dose–response relationship, with greater feature engagement linked to higher abstinence rates (Zhou et al., 2024). Systematic reviews also highlight that self-monitoring, personalisation, and well-designed reminders are key to driving engagement and effectiveness in digital health interventions (Rhodes et al., 2020; Szinay et al., 2020; Szinay et al., 2021). Moreover, features such as social influence, perceived utility, and techniques like facilitating self-recording and providing performance feedback contribute to app popularity and sustained user engagement (Crane et al., 2015; Szinay et al., 2020). While a dose–response relationship has not been established for sun protection interventions (Sheeran et al., 2020), some mobile apps demonstrate promise in improving sun safety behaviours (Buller et al., 2015).

The most frequently used BCTs were ‘instruction on how to perform a behaviour’, ‘information about health consequences’, ‘prompts/cues’, ‘feedback on outcomes of behaviour’, and ‘feedback on behaviour’. While the apps used several BCTs, there is an opportunity for improvement, as nine key BCTs identified in prior studies, including ‘social comparison’ and ‘demonstration of behaviour’, were not incorporated. Despite extensive research on self-regulatory techniques, such as feedback, action planning, and self-monitoring (Michie et al., 2009; Sheeran et al., 2020), effective self-regulatory BCTs were only sporadically utilised in the reviewed sun protection apps. Furthermore, BCTs linked to social influences on behaviour, which have proven effective for promoting sun protection (Rodrigues et al., 2013; Sheeran et al., 2020), were also notably absent from these applications.

In Part 2, participants reported an increase in self-regulatory sun protection skills as a result of using the app, particularly for planning activities outdoors to avoid midday sun. However, this skill seems to be situational. UK guidance outlines that individuals should use sun protection throughout March to October in the UK, advising individuals to seek shade from 11am to 3pm (Cancer Research UK, 2023). Part 2 indicated that participants were largely unaware of this guidance and primarily used the apps while abroad. The participants used the apps from July to October, when sun protection behaviours are most needed, but suggested that the app would not be useful in the UK during this time.

Qualitative analysis from Part 2 indicated that users valued aesthetics and a visually appealing design, echoing the findings from part 1 that many apps lack engaging features. The lowest ratings were for the domain of engagement, suggesting users desired greater functionality, including interactive and customisable features. In Part 2, participants highlighted the need for notifications, expressing a preference for reminders to reapply sunscreen and alerts when local UV levels increased. This feedback demonstrates a clear connection to the BCTs identified in Part 1, reinforcing the need for features that promote ongoing engagement and self-regulation. Previous research has found that participants would prefer more notifications in sun protection apps (Rodrigues et al., 2017, 2018). The SunSmart Global UV app, endorsed by the World Health Organisation and featuring UV monitoring with customisable notifications and tested usability (Hacker et al., 2018; Nicholson et al., 2019), exemplifies an app designed to meet these user preferences. Although it was not part of our study (i.e. unavailable on the app store during evaluation), its features align closely with the needs expressed by participants in Part 2, emphasising the value of customisable notifications for enhancing user engagement. However, the present study has also shown that there are individual differences involved with preference and opinions on notifications are mixed. Therefore, it may be useful for future apps to include settings to customise notification settings (Rodrigues et al., 2017, 2018), so users can choose how often they are notified in a day.

While both studies found that simplicity in app design and ease of use (e.g. account setup) were essential to ongoing engagement, Part 2 underscored that maintaining user engagement long-term requires enhancing motivational aspects – such as through gamification or interactive content, which were less emphasised in Part 1's assessments.

### Recommendations for sun protection apps development

Our findings highlight valuable opportunities for enhancing sun protection apps, focusing on the potential for theoretical and evidence-based input, improved technical functionality, and enriched aesthetics, interactivity, and personalisation features. Firstly, developers can significantly benefit from utilising established theories to inform their selection of features, particularly BCTs. Expanding the use of evidence could be accomplished by presenting compelling data on BCTs they adopt. Our findings resonate with prior research, underscoring the exciting potential for advancing the scientific evaluation of existing apps (Modave et al., 2015). To examine the impact of BCTs and other app characteristics, rigorous experimental studies using factorial designs should be conducted to assess how specific BCTs influence user behaviour across different populations and settings, allowing for more effective real-world applications.

Secondly, integrating location services within apps presents a favourable opportunity, as demonstrated by user feedback in this study. Participants found location services particularly beneficial for delivering real-time UV information tailored to their surroundings. Incorporating these services could include alerts based on local UV levels, prompting users to take protective action when needed. Additionally, participants appreciated notifications, suggesting that incorporating features for smartwatches could enhance access to sun protection advice on busy days (e.g. integrating real-time feedback and location-based notifications). Thirdly, to shape users’ knowledge apps can adopt gamification strategies, e.g. quizzes, crosswords, wordsearches and minigames, making content related to sun protection knowledge more engaging. Fourthly, personalisation features can greatly enhance user experience. Users expressed enthusiasm for guidance on optimal SPF based on individual skin types, as well as tailored recommendations for sunscreen application based on height and weight. Personalisation could also extend to sun protection advice tailored to users’ activity levels, geographic location, SPF preferences for different body areas, and even their personal/family history of skin cancer. Finally, offering most content for free while minimising the need for additional devices, along with promoting affordable sunscreen options, can greatly enhance user accessibility.

While the creative input from nonexperts in developing sun protection apps brings design advantages, collaboration with various experts (e.g. dermatologists, behavioural scientists) is indispensable to bolster the credibility of such applications. Evidence suggests that the engagement of experts is linked to the likelihood of increased app downloads (Pereira-Azevedo et al., 2016). For individuals looking for sun protection recommendations and practitioners offering advice on sun-safety, apps that track real-time UV levels, offer personalised recommendations based on individual skin types and activity levels, and monitor sun exposure are highly recommended.

### Strengths and limitations

This study makes a valuable contribution to the existing literature by systematically assessing the content of commercially available sun protection apps. Its key strengths lie in a comprehensive approach to identifying relevant apps and evaluating their content, complemented by user feedback on the top-rated apps. By conducting the studies separately, we were able to maintain a focused examination of each aspect, ultimately strengthening the findings. However, there are some limitations to note. First, while our search strategy for part 1 aimed to identify a broad range of sun protection apps using terms such as sun protection, UV index, and sunscreen, it did not include terms explicitly referencing skin cancer or melanoma. As a result, apps focused more specifically on skin cancer prevention or medical management may not have been captured. Future research could expand the search strategy to include terms such as skin cancer, melanoma, or skin cancer prevention to ensure the inclusion of apps that explicitly address these areas. This could provide a more comprehensive understanding of the digital tools available for skin cancer prevention and self-management. Second, in Part 2, a two-week longitudinal design was employed to understand user engagement with sun protection apps over time and to capture app engagement in typical UK conditions. User engagement with sun protection apps may have been influenced by weather conditions, with some participants mentioning reduced app usage on cloudy or colder days, highlighting the need for improved risk communication regarding sun protection in the UK. Although participants were asked about upcoming holidays in sunnier climates, the study did not systematically collect follow-up data to confirm if or when these vacations occurred. Additionally, daily weather or UVI conditions were not systematically tracked during the study period, which limits our understanding of how environmental factors impacted engagement. Future research should address these limitations by replicating the user-centred study incorporating controlled data on weather, UVI levels, and travel to sunnier locations to better understand app usage under varying conditions.

Third, the participant sample in Part 2 was primarily composed of UK-based, white women, many of whom had fair skin prone to burning and frequently travelled on holidays. While this demographic is relevant to high-risk populations for skin cancer, such as those with increased susceptibility to sunburn, the lack of representation from more diverse groups, including individuals with a family history of melanoma, extensive sun damage, or different cultural backgrounds, limits the generalisability of the findings. Future research should aim to include more diverse populations (e.g. personal or family history of skin cancer, tanning bed use) and explore cross-cultural comparisons to validate and extend these findings.

Finally, the two-year interval between the app content assessment (part 1) and user-centred study (part 2), primarily due to resource constraints, may have affected the availability, functionality, or relevance of some apps, as updates or removals occurred during this period. However, efforts were made to ensure that Part 2 focused on apps that remained accessible and relevant, with IONIQ Skincare included due to its availability, despite comparable scores to unavailable apps. Future studies should aim to minimise such intervals by aligning evaluations more closely in time and continuously monitoring app updates to capture changes in usability and content. When selecting an app, users should also consider the credibility of the information sources. Apps that are endorsed by or incorporate recommendations from dermatologists, behavioural science experts, health organisations, or link to trusted academic or professional sources offer added reassurance of their reliability and provide scientifically based, trustworthy advice.

## Conclusions

This study presents a comprehensive content analysis and user-centred evaluation of commercially available sun protection apps, identifying 48 apps and assessing its features and BCTs. Our findings highlight that sun protection apps designed to enhance knowledge, prioritise ease of use, and incorporate tailored, proactive features could promote sustained engagement. However, only a limited number of apps currently a comprehensive self-management approach to sun protection incorporating evidence-based strategies. Notably, the top-rated apps (i.e. REAPPLY and Qsun) demonstrated effective use of relevant BCTs and personalised features, showcasing promising models for future app development.

## Supplementary Material

Supplemental materials_02102024.docx

## Data Availability

Data available on reasonable request through the corresponding author (AMR).
