# Supplementary material for: Evaluating mobile apps for sun protection: content analysis and user preferences in a two-part study
Source: Health Psychol Behav Med. 2025 Jan 27;13(1):2456659. doi: 10.1080/21642850.2025.2456659 (PMC11774169; doi:10.1080/21642850.2025.2456659)
Supplement: Supplemental materials_02102024.docx [file RHPB_A_2456659_SM8079.docx]

**Supplemental Materials**

**Table 1.** Development of Feature Assessment Coding Criteria

| **Assessment Criteria** | **Rationale for Inclusion** | **Coding Criteria** |
| --- | --- | --- |
| Theory | Incorporated to ascertain whether there were examples of any theory that informed the development of the app which was available for the user to acknowledge | Theory present or not |
| Evidence | Evidence was included to determine if there were any reference to empirical evidence about behaviour change | Evidence Present or not |
| Tracking Type | Included to identify apps which tracked the relevant sun protection behaviours and support for behaviour change. | Sunscreen application, Sunbathing, UV index |
| Visualisation Features | Tracking features included within this assessment are evidenced as key components in enabling users to adopt sun protection behaviours | Photo Uploads, UV Photos, Skin Aging (face), UV Index Visualisations, Mole Visualisations |
| Social Media | Included in relation to evidence for social support | Social media incorporated or not |
| Location Features | Features relating to location that were not directly related to potential tailored features were included to understand scope of location features available. | Environment type, Sun/Clouds, UV Forecast  Current location and adjustable location features were not scored within the location feature criteria due to them being relevant to tailoring, therefore avoiding the potential of double coding a specific feature |
| Notifications | Notification feature criteria were included to ascertain whether the apps had the ability to send the user notifications relevant to the behaviour being tracked. Included in relation to evidence regarding prompts/cues | Notification features are present or not. |
| Tailoring | Tailoring Criteria was included to determine whether apps allowed the user to tailoring some or all features to their needs. Included in relation to evidence in relation to personalisation/tailoring | Location (adjustable, current), Skin colour, Tan, Burn, Sensitivity, UV Exposure, Clothing, Freckles, Hair colour, Eye colour, SPF, Height, Weight, Age, Gender  Criteria also included all aspects relating to the users’ personal characteristics (height, weight, gender, hair/eye colour etc). Despite these characteristics relating towards app personalisation, the researchers determined that personalisation features ultimately fall within the tailoring category. |
| Presence of Evidence-Based BCTs | Included due to BCTs already evidenced as effective | Initially, we had separate criteria for all BCTs identified in the app and 'effective BCTs' identified from previous literature (n=16). However, due to the majority of the BCTs identified within the apps falling into the 'effective BCT' category and were therefore scored twice, we decided to only include the effective BCT criteria to prevent overemphasising certain apps through scoring. |

**Table 2.** Full frequency mapping of evidence-based BCTs displayed in sun protection apps

| **Behaviour Change**  **Techniques^a^**  **Sun**  **Protection**  **App^b^** | **Number of BCTs^c^** | **4.1*** | **5.1*** | **7.1*** | **2.2*** | **2.3*** | **5.3*** | **2.7** | **9.1** | **1.1** | **1.4** | **2.1** | **2.4** |
| --- | --- | --- | --- | --- | --- | --- | --- | --- | --- | --- | --- | --- | --- |
| REAPPLY: Sunscreen Timekeeper | 5 | X | X |  | X |  | X |  | X |  |  |  |  |
| Qsun - Vitamin D & UV Tracker | 4 | X | X | X |  |  |  | X |  |  |  |  |  |
| UV Index Forecast | 4 | X | X | X |  |  |  | X |  |  |  |  |  |
| IONIQ Skincare | 3 | X | X | X |  |  |  |  |  |  |  |  |  |
| UVLens - UV Index | 3 |  |  | X | X | X |  |  |  |  |  |  |  |
| SunSafe | 3 | X | X | X |  |  |  |  |  |  |  |  |  |
| SunBlock - Protect your skin | 3 |  |  | X | X |  |  |  | X |  |  |  |  |
| Sunscreen Helper | 3 | X | X | X |  |  |  |  |  |  |  |  |  |
| Solarize | 3 | X | X | X |  |  |  |  |  |  |  |  |  |
| Uvlower | 3 |  |  | X | X | X |  |  |  |  |  |  |  |
| Sunface - UV - Selfie | 2 | X | X |  |  |  |  |  |  |  |  |  |  |
| My SKIN TRACK UV | 2 | X | X |  |  |  |  |  |  |  |  |  |  |
| UV Skin Protection | 2 | X | X |  |  |  |  |  |  |  |  |  |  |
| Save your skin | 2 | X | X |  |  |  |  |  |  |  |  |  |  |
| SkinSmart | 2 |  |  |  |  |  |  |  |  | X |  |  | X |
| Suncap - UV Index | 2 | X | X |  |  |  |  |  |  |  |  |  |  |
| SunDay: Vitamin D & UV Tracker | 2 | X |  |  |  |  |  |  |  |  |  | X |  |
| Sunscreen - Protect your skin | 2 | X | X |  |  |  |  |  |  |  |  |  |  |
| SunSense | 2 | X |  |  |  |  |  |  |  |  | X |  |  |
| UV Index Global | 2 | X | X |  |  |  |  |  |  |  |  |  |  |
| UV Index Now - UVI Mate | 2 |  |  | X |  |  |  | X |  |  |  |  |  |
| UV Index Widget | 2 | X |  |  |  |  |  | X |  |  |  |  |  |
| UV Notifier | 2 | X |  |  |  |  |  | X |  |  |  |  |  |
| UV Safe - Sun Protection | 2 | X |  | X |  |  |  |  |  |  |  |  |  |
| UV-INDEKS | 2 | X |  |  |  |  |  | X |  |  |  |  |  |
| Wear Sunscreen | 2 | X |  |  | X |  |  |  |  |  |  |  |  |
| AM Sun Expert | 2 |  |  |  |  | X |  | X |  |  |  |  |  |
| How to prevent a sunburn | 2 | X | X |  |  |  |  |  |  |  |  |  |  |
| DermIA | 1 |  |  | X |  |  |  |  |  |  |  |  |  |
| Mollie's fund | 1 | X |  |  |  |  |  |  |  |  |  |  |  |
| Cache Cache Soleil | 1 | X |  |  |  |  |  |  |  |  |  |  |  |
| IndiceUV | 1 | X |  |  |  |  |  |  |  |  |  |  |  |
| InfoSun | 1 | X |  |  |  |  |  |  |  |  |  |  |  |
| My UV Index | 1 | X |  |  |  |  |  |  |  |  |  |  |  |
| Sunbathing & UV | 1 | X |  |  |  |  |  |  |  |  |  |  |  |
| Sunbeam: UV Forecast | 1 |  |  |  |  |  |  | X |  |  |  |  |  |
| Sunny@SG | 1 | X |  |  |  |  |  |  |  |  |  |  |  |
| Sunscreenr Mobile | 1 |  |  | X |  |  |  |  |  |  |  |  |  |
| UV Index, Patrick Gludcelli | 1 | X |  |  |  |  |  |  |  |  |  |  |  |
| UV Index by dnzh | 1 | X |  |  |  |  |  |  |  |  |  |  |  |
| UV-Index, Inmeta | 1 | X |  |  |  |  |  |  |  |  |  |  |  |
| VBS UV Index Monitor | 1 |  |  | X |  |  |  |  |  |  |  |  |  |
| GlobalUV | 0 |  |  |  |  |  |  |  |  |  |  |  |  |
| Healthy Sun - safe tan | 0 |  |  |  |  |  |  |  |  |  |  |  |  |
| OzSun UV Alert | 0 |  |  |  |  |  |  |  |  |  |  |  |  |
| Sun Visor | 0 |  |  |  |  |  |  |  |  |  |  |  |  |
| UV Index - App | 0 |  |  |  |  |  |  |  |  |  |  |  |  |
| Uvisio | 0 |  |  |  |  |  |  |  |  |  |  |  |  |
|  | **Total** | 32 | 14 | 14 | 6 | 3 | 1 | 7 | 2 | 1 | 1 | 1 | 1 |

^a^Bold line indicates median number of BCTs.

# **Study 2 Initial Interview Schedule**

Think-aloud and semi-structured interview script

BEFORE RECORDING –

“During this session, you will spend some time using a sun protection app – of which you have been randomly allocated to. I would like you to ‘think aloud’ during this. This means trying to say everything that goes through your mind. This can be strange at first so we will begin with a practice task. I would like you to change the ringtone on your phone whilst saying everything you are thinking.”

ASK THEM TO DOWNLOAD THE APP

“The next part of this session will be recorded. This will be deleted as soon as transcription has taken place. Is that okay with you?”.

START RECORDING –

“Before we begin, do you consent to taking part in this study?”

“Thank you. In this study, we are investigating the usability of sun protection apps and the ways in which they can be improved in order to increase their engagement. I would like you to explore all of the different features the app you have been randomly allocated to has to offer. Whilst doing so, please try to say everything that you are thinking – including any criticisms. I am not the creator of this app so won’t be offended. I would also like to remind you that this is not a test, but simply a task to see how this app works for you. You are able to stop the study at any time, if you wish to do so. Please let me know when you feel as though you have explored all of the features.”

- Prompt the participant if they fall silent (‘What are you thinking?’).

Once they are done looking at the app, ask the following …

- “Did you think the app was easy to download and set up?”
- “Do you think this app was engaging?”
- If needed – “You mentioned ___. Could you please elaborate on that?”

“That is everything – thank you! We will be in contact in 2 weeks to arrange your online interview. In the meantime, please continue to use this app”.

STOP RECORDING

# **Study 2 Follow-up Interview Schedule**

START RECORDING –

“Before we begin, do you consent to taking part in this study?”

“Thank you.

- Please confirm which app you have tried.
- What did you like most about the app? Why?
- What would you change about the app if you could, and why?
- Do you think enough information was included in the app about how to protect yourself from harmful sun exposure?
- Would you consider continuing to use the app after today?
- (Only ask if they have previously used another sun protection app – this can be checked on Qualtrics) … You mentioned in the questionnaire attached to the recruitment post that you have previously used a sun protection app. How did that compare to the one you have been using in the past 2 weeks?
- Which feature of the app did you think was the most useful to help promote sun protection behaviours?
- Was the app easy to use? How did you find the font style and size and the layout?
- Were the different features of the app easy to find?
- Would you prefer using the app if it was more/less personalised?
- How likely are you to recommend this app to a friend or family member?
- Did you notice any medical terms in the app which were unfamiliar to you?
- Do you think using this app helped to increase your sun protection behaviours?
- Do you anticipate any advantages/disadvantages of using this app in your daily life?

“Thank you. That is all of the questions.”

STOP RECORDING
